# Impact of tariff on Indian AYUSH products: Evidence from top importers of India

**DOI:** 10.1016/j.jaim.2026.101329

**Published:** 2026-05-23

**Authors:** Saravanan Sengunthar, Diganta Sarkar, Pravin Jadhav

**Affiliations:** Institute of Infrastructure, Technology, Research and Management (IITRAM), Ahmedabad 380026, Gujarat, India

**Keywords:** Tariff reduction, Trade creation, Trade diversion, AYUSH products, Partial equilibrium analysis, Trade intelligence and negotiation advisor (TINA), Economic welfare, Export competitiveness, Policy implications

## Abstract

**Background** The global demand for Indian AYUSH (Ayurveda, Yoga & Naturopathy, Unani, Siddha, and Homeopathy) products has been rising, yet the impact of tariff reduction on their trade remains largely unexplored.

**Objective** This study aims to assess the effects of tariff reduction on AYUSH product exports to eight major importing countries, which collectively account for approximately 50% of India's AYUSH exports.

**Methods** The analysis covers 19 eight-digit HS codes specified by AYUSHEXCIL, which include traditional AYUSH products along with related cosmetic and dental preparations that contain herbal and traditional medicine ingredients, as dedicated six-digit HS codes for pure AYUSH products are not available in the international trade classification system. Using secondary trade data, the research employs a Partial Equilibrium Analysis through the Trade Intelligence and Negotiation Advisor (TINA) model to simulate trade scenarios under reduced tariff conditions.

**Results** The findings indicate that while tariff reductions generally enhance trade, a blanket zero-tariff policy across all AYUSH products is not advisable. In certain product categories, trade diversion outweighs trade creation, leading to potential welfare losses for India.

**Conclusion** The study provides critical insights for policymakers, suggesting a selective tariff reduction strategy that maximizes trade benefits while safeguarding national economic interests.The findings underscore the importance of integrating product-specific competitiveness, regulatory harmonization, and upstream value chain considerations into India's AYUSH export strategy

## 1 Introduction

In present time, the growing adoption of protectionist policies has substantially influenced global trade dynamics, with tariffs serving as a primary mechanism for governments to safeguard domestic industries from external competition. A tariff is a government-imposed tax on imports, aimed at regulating trade, protecting domestic industries, and generating revenue. However, the protectionist measures and trade barriers can impose substantial societal costs and losses, they also play a crucial role in safeguarding national security, supporting domestic producers, protecting the environment, and preserving market competition [1]. The country's export performance adversely affects, primarily due to the impact of tariff rates. Recently, the United States announced an additional 25% ad valorem tariff on specified Indian-origin goods in August 2025, which materially shifts in several key markets for AYUSH products and raises effective duty burdens on targeted lines and complicates exporter pricing and welfare assessments. By contrast, the UAE–India CEPA (Comprehensive Economic Partnership Agreement) continues to anchor zero or phased-down tariffs on many relevant HS lines[2].

To effectively enhance the trade and reduce/remove the barriers to trade and investment countries/government sign treaty which is known as Free Trade Agreement (FTA). When two or more governments define the rules of trade for all the participating signatories, it is known as Regional Trade Agreement (RTA). The effects of RTAs, particularly tariff reductions, can contribute to trade creation, especially benefiting low-income countries [3]. Cooperative efforts between partner countries can lead to more efficient trade volumes.

For example, Australia-China Free Trade Agreement (ACFTA) has demonstrated substantial economic and trade benefits for the two participating countries [4]. Similarly, Baier and Bergstrand [5] had proved how FTA actually increase member country's international trade.

Various regulatory measures act as barriers to trade. Medicinal products must comply with numerous stringent criteria to meet international trade standards. The challenges faced by Indian traditional medicine in international trade include regulatory hurdles that impede the registration and approval of traditional medicine products in foreign markets, particularly due to divergent safety and efficacy standards [6]. Understanding the different regulatory requirements of different countries is a tedious task [7]. However, meeting of requirement throughout all the countries, adhering to quality will increase the export of Indian traditional medicines.

As per WHO (2019) report [8], in underdeveloped countries, 80 percent of the population relies on traditional methods for primary healthcare which cause less side effects and is very easily available locally with very cheap price [9]. Herbal medicines have a long and storied history, with evidence of their use and documentation in various ancient medicinal systems, including those of India, China, Egypt, Greece, and Rome, dating back approximately 5000 years [10]. India holds the distinction of being the origin of some of the world's oldest traditional, complementary, and alternative systems of medicine, including Ayurveda, Siddha, Unani, Homeopathy, Yoga, and Naturopathy [11]. These systems reflect India's rich heritage of medicinal knowledge and practices [12]. Medicinal plants and certain minerals are essential components of traditional medicine. The country is endowed with remarkable biodiversity, particularly in medicinal plants [13] and medicinal knowledge detailed on Ayurveda [14]. According to the Red Data Book, India has the largest reserve of medicinal plants, with 7500 out of the approximately 17,000 plant species are known as medicinal purposes [15]. India can benefit from becoming a major hub for herbal medicine manufacturers. Acknowledging the strengths of traditional medicine, the Government of India has introduced the AYUSH initiative, an acronym that refers to Ayurveda, Yoga and Naturopathy, Unani, Siddha, and Homeopathy. The AYUSH mission focuses on the promotion and development of India's traditional medical systems and streams of **life-ministry** not only nationally but also globally, boosting and encouraging the herbal medicine industry, and preserving ancient evolutions of healthy lifestyles.

It is important to note that the number of recognized medicinal plant species in India differs across sources and time periods. For example, the Red Data Book listed 7500 medicinal species [15], while later studies such as Sen et al. [16] identified approximately 3000 species and recently Biological Survey of India (BSI), a total of 1915 medicinal species have been documented under the refined category. This discrepancy arises due to changes in taxonomic classification, updates in field research, and narrowing of definitions used for ‘medicinal’ categorization. These shifts reflect evolving understanding rather than loss of data or resources.

Since mid-2024, tariff policy has shifted materially in several key markets for AYUSH products. Notably, the United States announced an additional 25% ad valorem tariff on specified Indian-origin goods in August 2025, implemented via U.S. Customs and Border Protection guidance, which raises effective duty burdens on targeted lines and complicates exporter pricing and welfare assessments during the study horizon. These reciprocal measures were framed as broader trade countermeasures and may persist during negotiations. By contrast, the UAE–India CEPA (in force since May 2022) continues to anchor zero or phased-down tariffs on many relevant HS lines in the GCC hub, supporting the study's recommendation to prioritize tariff-creating markets in West Asia.

## 2 Global demand of AYUSH

The traditional and complementary medicine market has experienced significant growth driven by a global resurgence in herbal medicine and fewer side effects, presenting promising economic opportunities in this sector [9,16]. As per the 2019 WHO report [8], the global acceptance of traditional and complementary medicine has seen a fourfold increase from 1999 to 2019, accompanied by a significant rise in the number of countries adopting national policies on these practices from 25 in 1999 to 98 in 2018 and the international trade in medicinal plants and related products, valued at $ 60 billion in 2010, is projected to reach $ 5000 billion ($5 trillion) by 2050. According to the 2023 report by the Research and Information System for Developing Countries, the growth performance of medicinal and aromatic plants, herbal medicines, extracts, plant derivatives, and supplements has been remarkable, with an annual growth rate of 7.4 percent, while the herbal pharmaceutical sector grew at a faster rate of 18.5 percent, achieving a market share of 14.1 percent during 2014–2020 in globally [17]. Several reports have been published highlighting the global resurgence in demand for herbal medicine. According to Fortune Business Insights (2023), the global herbal medicine market size was $216.40 billion in 2023 and is projected to grow from $233.08 billion in 2024 to $437 billion by 2032 [18]. However, the projected growth in the US market is estimated to reach $37.90 billion by 2032, while the EU market currently dominates 44.82% of the global herbal medicine market in 2023. Similarly, global market size of herbal medicine was $166 billion in 2021 and is forecasted to reach $348 billion by 2028 [19]. Recently a study was conducted by IMARC group, the global Ayurvedic market was valued at $83 billion in 2021 and is projected to reach $128 billion by 2027, growing at a compound annual growth rate of 8.9% during the forecast period of 2022-2027. Similarly, the Indian ayurvedic product market reached Rs.748.5 billion in the year of 2023 and forecasted to reached Rs. 3207.6 billion by 2032 due to increasing of chronic diseases, various medical disorders and health consciousness among consumers [20]. Global consumers are benefiting through natural and traditional medical systems, driven by concerns over the adverse effects of synthetic drugs and a rising preference for holistic healthcare approaches [21] so the use of herbal medicines and other related products are growing exponentially all over the world [22]. The renewed interest in herbal and traditional medicine systems over the past few decades has driven the rapid expansion of the herbal sector, necessitating a heightened demand for medicinal plants to cater to the increasing global consumer preference for natural and holistic healthcare solutions [23].

While various sources project the value and growth of the global AYUSH and herbal medicine market, it is important to recognize that the figures vary significantly across institutions due to differences in definitional scope, forecasting methodologies, and product coverage. For instance, some reports refer to the broader herbal or traditional medicine market, including dietary supplements, wellness products, and nutraceuticals, while others focus strictly on codified medicinal systems, such as Ayurveda or Unani. As a result, agencies like WHO and RIS project the global herbal market to reach trillions of USD by 2050 based on long-term global health trends and demographic shifts[[8], [17]], whereas commercial reports such as Fortune Business Insights or IMARC offer more mid-term estimates (e.g., $233 billion to $437 billion by 2032) rooted in narrower product categories or specific geographies [[18], [20]]. However, the public health and prevention sector expanded from $328 billion in 2017 to $375 billion in 2020, fuelled by heightened investments during the COVID-19 crisis, accounting for 4% of global health expenditure $ 8800 ($ 8.8 trillion). Lessons from the pandemic emphasize the need for robust public health systems, with forecasts indicating a 5% annual growth rate, reaching $478 billion by 2025 [24]. Although the values may differ, they are not necessarily contradictory. Each projection reflects a different segment or analytical horizon within the larger traditional and complementary medicine industry. The diversity of estimates underscores the growing but still consolidating nature of global AYUSH trade data.

## 3 Economic opportunities for India

Traditional medicine not only serves as an economic resource but also provides holistic health security to a substantial segment of the Indian population and globally [25]. Due to the global resurgence of herbal medicine, the world herbal trade stands at $ 120 billion and is expected to reach $ 5000 billion ($ 5 trillion) by 2050 [25]. The global herbal medicine market is estimated to be worth $ 62 billion. The European Union dominates this market, accounting for 45% of the total. North America holds 11% of the market share, followed by Japan at 16% and the ASEAN countries at 19%. The remaining 4.1% of the global herbal market is attributed to the rest of the European Union [26]. In terms of global exports, China holds a significant share of 28%, while India's contribution is relatively lower at only 8.13% [16].

**Fig. 1:**
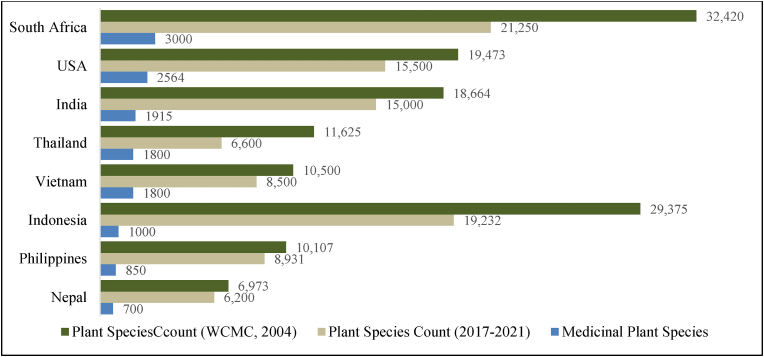
Worldwide medicinal plants species Utilisation.

Source: ([[16], [29], [30], [31], [32], [33], [34], [35], [36]])

Although demand for traditional medicine is increasing globally, despite India's vast reserves of biodiversity and long-standing traditional practices, Indian Export of Traditional Medicine is relatively less compared to the growing global demand. This demand presents a substantial opportunity for the Indian economy and potential to benefit multiple sectors, including agriculture, manufacturing, MSMEs, and the herbal healthcare industry, thereby generating significant economic prospects. The backward and forward linkages associated with the herbal manufacturing sector have significantly contributed to the growth of Indian agribusiness, entrepreneurship, and related industries [27]. The Ayurvedic industry encounters significant opportunities to establish itself within the global healthcare landscape, driven by the growing interest in holistic and natural healing systems that create a conducive environment for its expanded reach and influence worldwide [28]. China possesses approximately 4941 medicinal plant species within its total of 26,092 species [16], whereas India contains 1915 medicinal plant species out of 18664 total plant species. Indonesia, Malaysia, and Nepal also harbour substantial plant biodiversity, though less than China and India [[29], [30]]. The global distribution of total plant and medicinal plant species is depicted in Fig. 1. Despite its rich natural resources, India currently holds a relatively small market share in the rapidly expanding global trade of herbal medicine.

## 4 Indian AYUSH export landscape

**Fig. 2:**
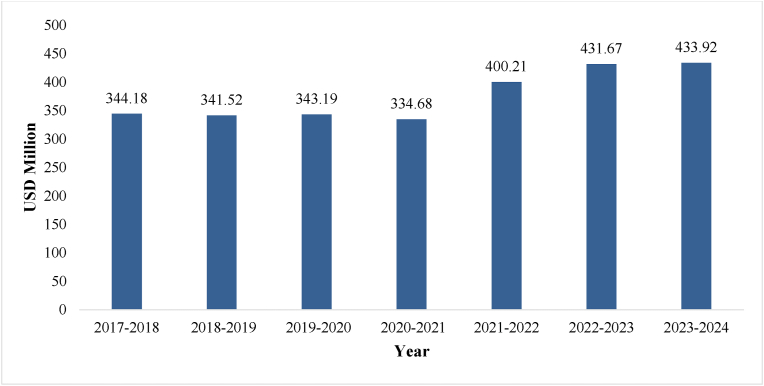
Year wise AYUSH export from India.

India has been exporting a wide range of AYUSH products, herbal extracts, and Ayurvedic formulations to various countries around the world. Accordingly, the Ministry of AYUSH, Government of India, has identified 19 types of products with 8-digit HS code (list of Supplementry file - Appendix A) which come under Ayurveda, Yoga and Naturopathy, Unani, Siddha, and Homeopathy. Fig. 2 illustrates the steady year-on-year growth in the export value of AYUSH products from India globally.

Source:DGFT, Ministry of Commerce and Industry, Government of India [37]

**Fig. 3:**
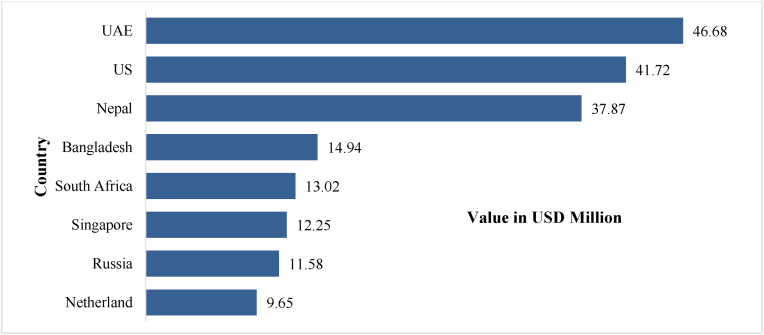
Amount of trade to top importer countries.

Source: DGFT, Ministry of Commerce and Industry, Government of India [37]

Fig. 3 illustrates the average import volume of AYUSH products from India over the past seven years. It highlights eight countries, which collectively account for nearly 50% of AYUSH imports from India during this period. These countries, therefore, serve as the most significant trade influencers in the global AYUSH market. The countries holding the top 3 positions are United Arab Emirates (UAE), United States (US) and Nepal respectively.

India's export strength in AYUSH products is largely built upon its robust domestic capacity for value addition rather than self-sufficiency in all raw inputs. A significant portion of inputs used in Ayurvedic and herbal formulations, such as crude herbs, botanicals, resins, and minerals, are imported from biodiversity-rich countries, including China, Nepal, and select Southeast Asian and African economies. These raw materials are then processed, standardized, and transformed into high-value medicaments and personal care products under the AYUSH umbrella before being exported to global markets. This import-export dynamic reflects India's strategic position in the global herbal value chain as a value-added exporter rather than a primary producer of all inputs. Therefore, any analysis of tariff impacts on AYUSH exports should be contextualized within this vertically integrated trade structure. Evaluating the effects of tariff reduction in isolation without accounting for upstream input dependencies risks overlooking key cost and competitiveness implications for Indian exporters.

## 5 Methodology

The study employs a quantitative, simulation-based approach to assess the impact of tariff reductions on Indian AYUSH product exports using the Trade Intelligence and Negotiation Adviser (TINA) software, developed by the trade policy and facilitation section of the trade, Investment and Innovation Division of the United Nation Economic and Social Commission for Asia and the Pacific [38]. TINA is designed to assist trade negotiation agreements and enhance trade. ESCAP provides insights on tariffs, non-tariff measures, agreements, and bilateral flows and serves to identify key products for better tariff negotiation.

The methodology is grounded in the framework of FTA negotiations and builds upon the partial equilibrium model of trade. The research design incorporates pooled secondary data and emphasizes an empirical evaluation of tariff effects on trade flows across eight top AYUSH Products importers from India.

The analytical foundation draws from standard FTA negotiation methodology, wherein tariff liberalization scenarios are simulated to assess the trade responsiveness of selected products. The prioritization of products for inclusion in simulated tariff negotiations was based on a three-step framework: identifying export potential, simulating tariff changes, and interpreting the results through a negotiation lens.

TINA was used to simulate the effects of removing or reducing tariffs for products across the selected countries. The model incorporates import demand and export supply elasticities sourced from relevant empirical literature [39,40]. For each product-country pair, the simulation estimated trade creation and trade diversion values based on changes in relative prices and the responsiveness of trade flows to tariff adjustments. All TINA filters were deliberately disabled during this process. The analysis was conducted using data from the year 2023, which reflects the most current trade and tariff patterns.

Finally, a prioritization matrix was derived from the simulation outputs to classify products into two categories: those recommended for tariff concessions and those for which tariff reduction was not advisable. The classification was based on whether trade creation outweighed trade diversion, aligning with the practical tariff line scheduling logic used in FTA negotiations. The emphasis was placed on maximizing economic welfare gains while minimizing the risk of inefficient trade deflection. This approach is consistent with the theoretical foundation laid in customs union analysis [41] and further operationalized through the partial equilibrium formulations [42,43]. The simulation results guided the policy recommendations regarding product-specific tariff liberalization strategies tailored to each of the eight major importing countries.

### 5.1 Data

As per AYUSHEXCIL, the export promotion council arm of the Ministry of AYUSH, Government of India, 19 HS 8-digit product codes have been identified as about raw materials and finished products of AYUSH [44]. In this study, the export data of these 19 products with an 8-digit HS code have been collected from a government source, namely, the Directorate General of Foreign Trade (DGFT), Ministry of Commerce and Industry, Government of India [37].

The World Customs Organization standardizes the numerical methods of international traded products in the form of the Harmonized System (HS), where the initial six digits are globally harmonized, while the final two digits are tailored for domestic categorization [45]. The 6-digit HS code is utilized in FTA negotiations owing to its international standardization. This ensures consistent classification across nations, while the 8-digit code incorporates country-specific extensions. Employing the 6-digit level facilitates trade negotiations by maintaining consistency, mitigating complexities stemming from national variations, and enabling greater flexibility in tariff commitments.

**Fig. 4:**
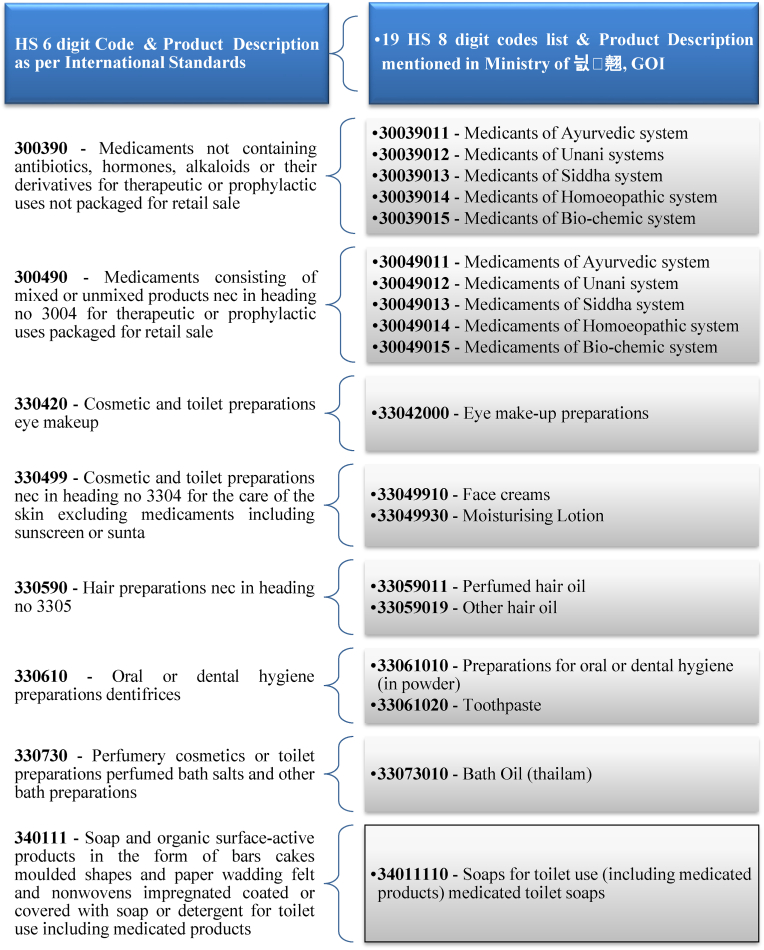
8-Digit to 6-digit HS code conversion Chart.

Source: [[37], [44], [45]]

Fig. 4 is an explanation of the conversion of HS 8-digit code 19 AYUSH products to HS 6-digit code 8 Products as per the H3 Nomenclature, Edition 2022 [45]. This is the most detailed level at which trade data is available and is the closest to India's HS eight-digit national tariff level. For six-digit HS commodities, we have only eight commodities out of nineteen AYUSH commodities, which come under six-digit HS commodities in detail.

While harmonizing the 8-digit national HS codes with the international 6-digit system, it is important to acknowledge the classification discrepancies that exist in the context of AYUSH product exports. The HS codes 30039011 to 30039015 and 30049011 to 30049015 are explicitly associated with the Ayurvedic, Unani, Siddha, Homeopathy, and Bio-chemic systems. These subcategories are embedded within the broader international HS codes 300390 and 300490, which pertain to medicaments not containing antibiotics or hormones. In contrast, the remaining HS codes used in this study are classified under cosmetics, dental hygiene, and bath products. Although these may not fall under traditional AYUSH classifications, these products fall under the broad category of Natural and herbal products, so these are included in the Ministry of AYUSH's official product list for export tracking purposes.

This inclusion is justified by practical trade practices, wherein exporters often classify AYUSH products, especially herbal formulations and plant-based personal care items, under closely related HS categories due to the absence of dedicated HS 6-digit codes for many AYUSH items in the global nomenclature. This data alignment, while slightly expanding beyond strictly defined traditional medicine codes, reflects the Ministry's pragmatic approach to product classification and export reporting. Therefore, any perceived overlap with cosmetic or hygiene product categories should be interpreted in light of the Indian government's operational classification, which aims to capture the breadth of AYUSH-related exports in the absence of globally harmonized coding structures.

### 5.2 Empirical strategy

The study employs a partial equilibrium framework using the Trade Intelligence and Negotiation Adviser (TINA) software [46]. This methodology is grounded in established approaches for conducting partial equilibrium analysis and integrates the authors' practical experience in formulating import demand elasticity within trade negotiations [[39], [40]]. Notably, while generating the negotiation list, all filters were skipped to ensure a comprehensive analysis. Additionally, the data for the year 2023 was utilized to build the negotiation list. The concepts of trade creation and trade diversion, which are central to the analysis of FTA negotiations, were originally introduced by Viner in 1950 [41].

#### 5.2.1 Trade creation

It is assumed that the trade creation effect refers to the rise in demand for commodity *i* in country *j* from exporting country *k*, driven by a reduction in price. This price decline occurs due to the removal or reduction of tariffs and non-tariff barriers, assuming complete price transmission. Trade creation refers to the expansion of trade volumes following trade liberalization, leading to the replacement of inefficient producers within a given preferential trade area. This study also adopts the approach of Laird & Yeats [43] in estimating trade creation, beginning with the formulation of simplified demand and supply functions alongside an equilibrium condition. For example, the following is a simplified import demand function for nation *j* from country *k* of commodity *i*:

(Eq. 1) **M_ijk_ = F(Y_j_, P_ij_, P_ik_)**

Where, M _ijk_ is the quantity of imports of product i by country j from country k, Y_j_ is the income level of the importing country j, P_ij_ is the domestic price of product i in country j, P_ik_ is the export price of product i from country k and the notation F( …) represents a functional relationship, indicating that import demand is determined by a combination of national income and price factors.

The export supply function of exporting nation *k* for commodity *i* can be written as follows:

(Eq. 2) **X _ijk_ = F (P_ikj_)**

Where X_ijk_ is the quantity of product i exported by country k to country j, P_ikj_ is the price received by exporter k when selling to country j and F(P_ikj_) implies that the export supply depends on the price received in the destination market.

Therefore, based on Equations (Eq. (1)) and (Eq. (2)), the equilibrium condition governing trade between countries *j* and *k* within the standard partial equilibrium framework can be expressed as follows:

(Eq. 3) ***M*_*ijk*_** = ***X*_*ikj*_**

In a free trade scenario, the exporting country *k*'s export price plus transportation and insurance costs will equal the domestic price of the commodity *i* in the importing market *j*. This means that the price will increase by the amount equal to the ad valorem incidence of any tariff or non-tariff distortion applied to the good. Thus:

(Eq. 4) **P_ijk_ = P_ikj_ (l + t_ijk_)**

Where P_ijk_ is the domestic price of product i in country j after the tariff is applied, t_ijk_ is the ad valorem tariff rate imposed by country j on imports from country k.

Therefore, total differentiation of domestic price (Eq. (4)) with respect to tariff and foreign price:

(Eq. 5) **dP_ijk_ = P_ikj_. dt_ijk_ + (l + t_ijk_). dP_ikj_**

Assuming a constant elasticity of import demand (Em), the percentage change in imports can be described as:

(Eq. 6) **dM_ijk_ /M_ijk_ = Em.(dP_ijk_ /P_ijk_)**

Substituting the expression for dP_ijk_ from Equation (5) into Equation (6) yields:

(Eq. 7) **dM_ijk_ /M_ijk_ = Em. (dt_ijk_ /(l + t_ijk_) + dP_ijk_ /P_ikj_)**

The export supply elasticity is expressed as:

(Eq. 8) **dP_ikj_ /P_ikj_ = (dX_ikj_ /X_ikj_)/Ex**

Where, Ex is the elasticity of export supply.

Using the equilibrium condition:

(Eq. 9) **dM_ijk_ /M_ijk_ = dX_ikj_ /X_ikj_**

By substituting (Eq. (9)) into (Eq. (8)) and subsequently incorporating the resulting expression into (Eq. (7)), we derive a formulation that facilitates the computation of the trade creation effect. Furthermore (Eq. (9)), represents the growth in exports of commodity *i* from exporting country *k* to importing country *j*. Consequently, the expression for trade creation can be formulated as follows:

(Eq. 10) **TC _ijk_ = M _ijk_ Em.dt _ijk_ /[(l + t _ijk_)].[1.(Em/Ex)]**

Where TC_ijk_ is the trade creation effect resulting from tariff reduction on i product in j country from country k, Em is the price elasticity of import demand, Ex is the price elasticity of export supply and dt_ijk_ is the change in tariff rate applied by country j to country k.

While the above captures trade creation from partner countries, it is equally important to account for the displacement of imports from more efficient non-partner sources, as explained through the trade diversion formula below.

#### 5.2.2 Trade diversion

When importers reallocate their sourcing of goods in response to variations in import prices from one supplier, while prices from alternative suppliers remain unchanged. Specifically, a decline in prices in a particular exporting country incentivizes importers to increase their purchases from that country while reducing imports from others whose prices remain stable. Additionally, trade diversion may occur not solely due to changes in export prices but also as a result of the introduction or removal of preferential trade agreements benefiting specific suppliers, while the treatment of other sources remains unaffected. Moreover, such diversion can stem from relative shifts in the regulatory or policy framework of the importing country, wherein differential changes in trade policies alter the competitive standing of various foreign suppliers.

A formulation created by Baldwin & Murray [42] makes it possible to calculate trade diversion even in cases where the elasticity of substitution between alternative providers is unknown. However, this method requires the ability to compute the degree of import penetration by non-preference-receiving nations, or the proportion of imports from these nations in apparent domestic consumption. The trade diversion (absence of explicit value for the elasticity of substitution) formula can be written as:

(Eq. 11) **TD_ijk_ = TC_ijk_.(Mn_ij_ /V_ij_)**

Where Mn_ij_ is the import of product I from non-preference countries into country j, V_ij_ is the total apparent consumption of product i in country j.

In the presence of explicitly defined values for the elasticity of substitution between goods from different sources, this elasticity can be formally characterized as the percentage change in the relative market shares of a product resulting from a one percent change in its relative prices across alternative sources. That is:

(Eq. 12) $$\mathrm{Es}=\frac{d\left(\Sigma M\;\mathrm{ijk}/\Sigma M\;\mathrm{ijK}\;\right)/\left(\Sigma M\;\mathrm{ijk}\;/\Sigma M\;\mathrm{ijK}\right)}{d\left(P\;\mathrm{ijk}\;/P\;\mathrm{ijK}\;\right)/\left(P\;\mathrm{ijk}\;/P\;\mathrm{ijK}\;\right)}$$

Where Es is the elasticity of substitution and P_ijk_ is the relative price of product i in country j from country k.

Note k denotes one group of foreign suppliers and K denotes the other group of foreign suppliers. The summation considers only across country group k or K, not across product i or importer j [43].

From the expression (Eq. (12)), systematic expansion, substitution, and algebraic manipulation, the resulting expression quantifies the change in imports from a specific country, representing trade diversion effects. Which arising from shifts in duty-paid prices relative to competing sources following a commercial policy adjustment. The formula can be written:

(Eq. 13) $$\mathrm{TD}\;\mathrm{ijk}=\frac{M_{\mathrm{ijk}}\;\Sigma M\;\mathrm{ijk}\;.\Sigma M\;\mathrm{ijK}.\mathrm{Es}\frac{d\left(P\;\mathrm{ijk}\;/P\;\mathrm{ijK}\;\right)}{P\;\mathrm{ijk}\;/P\;\mathrm{ijK}}}{{\Sigma M}_{\mathrm{ijk}}\Sigma M\;\mathrm{ijk}+\Sigma M\;\mathrm{ijK}+\Sigma M\;\mathrm{ijk}\;.\mathrm{Es}\frac{d\left(P\;\mathrm{ijk}\;/P\;\mathrm{ijK}\;\right)}{P\;\mathrm{ijk}\;/P\;\mathrm{ijK}}}$$

In particular, the trade diversion states import shift from a more efficient non-member country to a more expensive (less efficient) member country due to preferential trade policies, leading to the reduction of global welfare. On the other hand, trade creation allows substitution of more expensive domestic production with cheaper imports from a member country, potentially lead to a gain global welfare. Which is summarised:

(Eq. 14) **W _ijk_ = 0.5(dt _ijk_. dM _ijk_)**

Where Wijk is the gain of global welfare due to import of i product in country j from country k and t_ijk_ is the tariff rate or non-tariff distortion of product i in county j from country k.

**Fig. 5:**
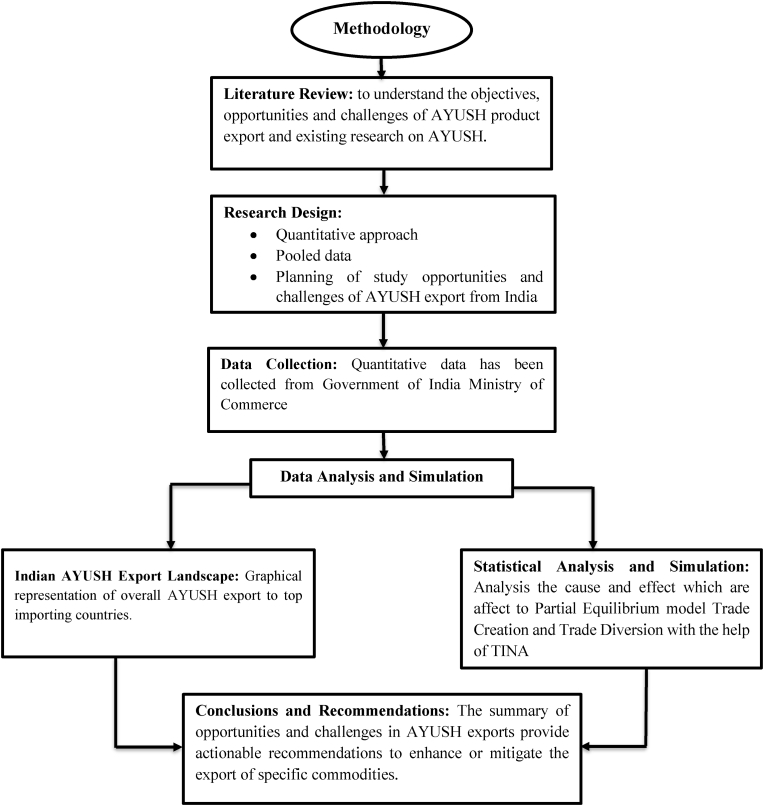
Summary of Methodology.

These insights can guide policy recommendations regarding the imposition of tariffs on AYUSH commodities. Fig. 5

## 6 Current tariff

The 6-digit HS code product-wise tariff rate on top export countries mentioned listed in Supplementry file (Appendix B), where Singapore imposes no tariffs on any of these products due to its Free Trade Agreement (FTA) with India, while the Netherland (European Union) has not levied any tariffs on Indian AYUSH products. Consequently, the tariff rates for these two countries are recorded as zero. Similarly, among the eight products analysed, the United States has imposed tariffs only on the product classified under HS code 330730. In contrast, the UAE and South Africa have applied tariffs on six products, except for those under HS codes 300390 and 300490. Russia has imposed tariffs on all products except for HS code 300390. However, both Nepal and Bangladesh have imposed tariffs on all 6-digit HS code AYUSH products.

## 7 Findings

Table 1 exhibits the results of the TINA simulation, which examined trade creation and trade diversion under tariff reduction scenario for FTA negotiations, are detailed in Supplementry file (Appendix C to H). Since there are currently no tariffs on any of the 6-digit HS codes, the tariff reduction simulation with Singapore and the Netherlands was deemed infeasible. These TINA simulation results should be interpreted with the 2025 policy updates in mind particularly U.S. reciprocal tariffs and UAE - CEPA preferences because trade creation/diversion magnitudes can shift at the margin when effective ad valorem incidence changes between partners.

**Table 1:** Trade creation and trade diversion due to FTA negotiation.

HS 6-digit Code		300390	300490	330420	330499	330590	330610	330730	340111
**UAE**	**TC**	NA	NA	19113	4138620	671944	162341	7599	2463483
	**TD**	NA	NA	43095	1360820	1111693	910250	12114	970998
	**PC**	NA	NA	10.25	28.32	10.52	7.77	11.51	21.58
**USA**	**TC**	NA	NA	NA	NA	NA	NA	42499	NA
	**TD**	NA	NA	NA	NA	NA	NA	41384	NA
	**PC**	NA	NA	NA	NA	NA	NA	15.36	NA
**Nepal**	**TC**	2231160	62418966	487705	62418966	18090366	1950510	19371	5585
	**TD**	13736	1557281	103817	1557281	586172	37593	4419	86449
	**PC**	9.01	37.18	58.92	56.78	141.73	40.45	59.76	3.72
**Bangladesh**	**TC**	1494220	3777039	19838	11341104	6330947	182803	135796	141356
	**TD**	355331	867455	45788	2741590	410129	200016	12459	351789
	**PC**	36.09	41.86	39.42	79.88	293.21	10.21	19.4	29.62
**South Africa**	**TC**	NA	NA	907	100335	146629	619792	14073	550594
	**TD**	NA	NA	2693	343986	192042	331611	1569	204106
	**PC**	NA	NA	33.4	32.05	43.24	37.47	248.88	90.38
**Russia**	**TC**	NA	361619799	1574	42915	55267	297907	NA	3483
	**TD**	NA	18301637	5669	99194	20562	292045	NA	4844
	**PC**	NA	64.46	11.69	13.09	33.73	17.98	NA	13.44

∗TC- Trade Creation, TD – Trade Diversion & PC – Percentage Change.

For the **UAE**, the analysis revealed that products with HS codes 330590, 330610, and 330730 experienced greater trade diversion than trade creation due to tariff reductions. The trade creation amounted to 6,621,216 USD, while trade diversion reached 4,408,970 USD. Notably, three items showed higher trade diversion than trade creation. The HS codes 300390 and 300490 currently have zero tariffs. Tariff reductions were recommended for HS codes 330420, 330499, and 340111, while reductions were not advised for HS codes 330590, 330610, and 330730.

In the case of the **US**, tariffs were imposed on only one product, resulting in greater trade creation (42,499 USD) than trade diversion (41,384 USD). No items exhibited higher trade diversion than trade creation. The HS codes 300390, 300490, 330420, 330499, 330590, 330610, and 340111 currently have zero tariffs. A tariff reduction was recommended for HS code 330730.

For **Nepal**, tariff reductions led to greater trade creation (147,617,044 USD) compared to trade diversion (3,860,299 USD) across all products. Only one item showed higher trade diversion than trade creation. No HS codes currently have zero tariffs. However, tariff reductions were recommended for HS codes 300390, 300490, 330420, 330499, 330590, 330610, and 330730. No HS codes were identified where tariff reductions were not recommended.

In **Bangladesh**, products with HS codes 330610 and 340111 exhibited higher trade diversion (4,386,964 USD) than trade creation (23,079,106 USD). Three items showed trade diversion surpassing trade creation. No HS codes currently have zero tariffs. Tariff reductions were advised for HS codes 300390, 300490, 330499, 330590, and 330730, while they were not recommended for HS codes 330420, 330610, and 340111.

For **South Africa**, tariff reductions led to greater trade diversion than trade creation for products with HS codes 330420 and 330499. The trade creation was 1,184,459 USD, while trade diversion was 537,286 USD. Three items exhibited higher trade diversion than trade creation. HS codes 300390 and 300490 currently have zero tariffs. Tariff reductions were recommended for HS codes 330610, 330730, and 340111, while they were not recommended for HS codes 330420, 330499, and 330590.

In **Russia**, products with HS codes 330420, 330499, and 340111 also experienced greater trade diversion than trade creation. The trade creation amounted to 2,151,462,275 USD, whereas trade diversion reached 186,142,244 USD. Three items showed higher trade diversion than trade creation. HS codes 300390 and 330730 currently have zero tariffs. Tariff reductions were recommended for HS codes 300490, 330590, and 330610, while they were not recommended for HS codes 330420, 330499, and 340111.

## 8 Discussion

The analysis of India's AYUSH product exports reveals substantial opportunities for enhancing market access and driving economic growth through targeted trade policy interventions. By leveraging insights from the TINA simulation, India can adopt tailored tariff reduction strategies that align with the unique market dynamics of its key trading partners, thereby maximizing trade creation while minimizing inefficiencies. This approach strengthens India's position as a global leader in the traditional medicine market [47].

A significant strength of India's AYUSH sector lies in its ability to capitalize on cultural and geographic synergies, particularly in markets like Nepal, where favourable trade creation dynamics indicate substantial untapped potential. Prioritizing tariff liberalization in such markets can boost export volumes and yield considerable welfare gains, reinforcing India's leadership in traditional medicine systems [48]. This focus on culturally aligned markets can serve as a blueprint for expanding India's global presence.

In US market, while many AYUSH products face low or zero MFN, the additional 25% reciprocal tariff implemented in August 2025, on specified Indian goods raises pricing and margin risks in product categories that fall under the AYUSH's scope. Exporters to quantify incidence and assess whether trade creation for HS 330730 in our simulation remains net-positive after the recent reciprocal tariff imposition [49].

However, a critical challenge persists in the regulatory misalignment of AYUSH products across many importing countries. In markets such as the United States and the European Union, AYUSH products are often not recognized as medicinal but are instead classified as nutraceuticals, dietary supplements, or cosmetics, subjecting them to distinct quality, labeling, and safety standards [[6], [50], [51]]. This reclassification complicates market access and requires exporters to adapt to alternative product categorizations, potentially diluting the identity and perceived value of AYUSH products. To address this, India should advocate for greater international recognition of AYUSH systems through bilateral trade agreements and collaboration with global frameworks like the WHO Traditional Medicine Strategy, which promotes the integration of traditional medicine into national health systems [47]. Such efforts can enhance the global legitimacy of AYUSH products and streamline export processes.

In markets like the UAE, South Africa, Bangladesh, and Russia, diverse trade outcomes highlight the importance of a nuanced, product-specific approach to tariff negotiations. By targeting product categories with high trade creation potential, India can optimize market access while addressing competitive challenges, ensuring that export growth is driven by quality and cost competitiveness for sustainable economic benefits. Whereas the US, with its favourable tariff environment for most AYUSH products, presents a unique opportunity to address non-tariff barriers. Prioritizing compliance with regulatory standards set by the United States Food and Drug Administration (FDA) can unlock further market potential and enhance the credibility of AYUSH products as high-quality offerings [52]. This focus on regulatory alignment can catalyse broader market penetration and support efforts to establish AYUSH as a recognized medicinal category. In the case of Bangladesh and South Africa, positive trade creation in specific product categories underscores the potential for targeted export promotion. By focusing on high-performing products and mitigating trade diversion risks, India can strengthen its market presence while ensuring efficient resource allocation. Similarly, in Russia, robust trade creation in certain product lines provides a foundation for strategic tariff diplomacy, complemented by efforts to enhance supply-side competitiveness. Overall, the findings advocate for a strategic, evidence-based approach to tariff policy that prioritizes product-level competitiveness and market-specific dynamics. By aligning export strategies with the regulatory and competitive landscapes of its trading partners and actively pursuing global recognition for AYUSH products, India can unlock significant trade potential for its AYUSH sector. Integrating regulatory recognition frameworks, such as WHO Traditional Medicine Strategy indicators, into future trade analyses will further refine policy recommendations, ensuring that India maximizes its net trade and welfare gains while solidifying its leadership in the global traditional medicine market [47].

## 9 Conclusion

The simulation results of TINA suggest tariff reduction recommendations across various countries, highlighting key distinctions in trade policy considerations. The total Trade Creation value for all 8 countries can be mentioned here. In the case of the UAE, tariff reductions are recommended for products with HS codes 330420, 330499, and 340111, whereas reductions are not advisable for HS codes 330590, 330610, and 330730. For the United States, the current tariff is zero for products under HS codes 300390, 300490, 330420, 330499, 330590, 330610, and 340111, with tariff reduction recommended only for HS code 330730. Nepal demonstrates a broader scope for tariff reductions, with recommendations for HS codes 300390, 300490, 330420, 330499, 330590, 330610, and 330730, while no products are categorized as unsuitable for tariff reductions. In Bangladesh, tariff reductions are suggested for HS codes 300390, 300490, 330499, 330590, and 330730, whereas they are not recommended for HS codes 330420, 330610, and 340111. Similarly, in South Africa, tariff reductions are advisable for HS codes 330610, 330730, and 340111, while HS codes 330420, 330499, and 330590 are deemed unsuitable for reductions. Lastly, in Russia, tariff reductions are recommended for HS codes 300490, 330590, and 330610, while HS codes 330420, 330499, and 340111 are identified as products where tariff reductions are not recommended.

Overall, the study highlights that tariff reductions must be targeted and evidence-driven. Broad-based tariff liberalization may not benefit all product segments equally, especially where trade diversion dominates. The findings underscore the importance of integrating product-specific competitiveness, regulatory harmonization, and upstream value chain considerations into India's AYUSH export strategy.

## 10 Limitation and way forward: harmonization of AYUSH product classification

A key consideration in this study pertains to the classification structure of AYUSH-related export products. Among the 19 HS 8-digit codes identified by the Ministry of AYUSH, only ten, ranging from 30039011 to 30039015 and 30049011 to 30049015, are explicitly and exclusively aligned with traditional medicinal systems such as Ayurveda, Unani, Siddha, Homeopathy, and Bio-chemic. The remaining codes, such as 33042000, 33049910, 33049930, 33059011, 33059019, 33061010, 33061020, 33073010, and 34011110, are currently categorized under cosmetics, dental hygiene, and bath preparations in the global HS nomenclature. These products, although herbal or plant-based in nature and included in the Ministry's export tracking list, are not uniformly recognized as traditional medicinal products in international classification systems.

To address this structural limitation and enhance the precision of tariff simulation analysis, there is a strong case for advocating the creation of dedicated 6-digit HS codes that distinctly represent AYUSH-based natural products. Such a measure would bring greater transparency and uniformity in classification, allowing trade analysts and policymakers to distinguish AYUSH exports more accurately from conventional cosmetic or personal care goods. This refinement would also align with the Ministry of AYUSH's broader objective of promoting traditional Indian medicine globally by enabling clearer tracking, targeted negotiations, and better-informed export promotion strategies.

## Author Contribution

SS devised the study, worked on data modelling and provided the tables. DS refined the draft and lead in responding to reviewer’s comments. PJ developed the policy recommendations.

## Declaration of generative AI in scientific writing

The authors have not used any generative AI tool to prepare the manuscript.

## Sources of funding

None

## Declaration of competing interest

The authors declare that they have no known competing financial interests or personal relationships that could have appeared to influence the work reported in this paper.
