# Supplementary material for: Impact of tariff on Indian AYUSH products: Evidence from top importers of India
Source: J Ayurveda Integr Med. 2026 May 23;17(3):101329. doi: 10.1016/j.jaim.2026.101329 (PMC13226236; doi:10.1016/j.jaim.2026.101329)
Supplement: Multimedia component 1 [file mmc1.docx]

| **Appendix. A: The 19 eight-digit HS codes specified by AYUSHEXCIL** | | |
| --- | --- | --- |
| **SL. No.** | **HS Code** | **Description** |
| 1 | 30039011 | Medicants of Ayurvedic system |
| 2 | 30039012 | Medicants of Unani systems |
| 3 | 30039013 | Medicants of Siddha system |
| 4 | 30039014 | Medicants of Homoeopathic system |
| 5 | 30039015 | Medicants of Bio-chemic system |
| 6 | 30049011 | Medicaments of Ayurvedic system |
| 7 | 30049012 | Medicaments of Unani system |
| 8 | 30049013 | Medicaments of Siddha system |
| 9 | 30049014 | Medicaments of Homoeopathic system |
| 10 | 30049015 | Medicaments of Bio-chemic system |
| 11 | 33042000 | Eye make-up preparations |
| 12 | 33049910 | Face creams |
| 13 | 33049930 | Moisturising Lotion |
| 14 | 33059011 | Perfumed hair oil |
| 15 | 33059019 | Other hair oil |
| 16 | 33061010 | Preparations for oral or dental hygiene (in powder) |
| 17 | 33061020 | Toothpaste |
| 18 | 33073010 | Bath Oil (thailam) |
| 19 | 34011110 | Soaps for toilet use (including medicated products) medicated toilet soaps |

| **Appendix. B: Product-wise Tariff on Top Export Countries** | | | | | | | | | |
| --- | --- | --- | --- | --- | --- | --- | --- | --- | --- |
| **HS 6-digit Code** | **Product Description** | **UAE** | **US** | **Nepal** | **Bangladesh** | **South Africa** | **Singapore** | **Russian Federation** | **Netherland** |
| **300390** | Medicaments not containing antibiotics, hormones, alkaloids or their derivatives for therapeutic or prophylactic uses not packaged for retail sale | 0.00 | 0.00 | 11.67 | 10.00 | 0.00 | 0.00 | 0.00 | 0.00 |
| **300490** | Medicaments consisting of mixed or unmixed products nec in heading no 3004 for therapeutic or prophylactic uses packaged for retail sale | 0.00 | 0.00 | 11.25 | 8.75 | 0.00 | 0.00 | 3.80 | 0.00 |
| **330420** | Cosmetic and toilet preparations eye makeup | 5.00 | 0.00 | 15.00 | 25.00 | 20.00 | 0.00 | 6.50 | 0.00 |
| **330499** | Cosmetic and toilet preparations nec in heading no 3304 for the care of the skin excluding medicaments including sunscreen or suntan preparations | 5.00 | 0.00 | 15.00 | 25.00 | 20.00 | 0.00 | 6.50 | 0.00 |
| **330590** | Hair preparations nec in heading no 3305 | 5.00 | 0.00 | 15.00 | 25.00 | 20.00 | 0.00 | 6.50 | 0.00 |
| **330610** | Oral or dental hygiene preparations dentifrices | 5.00 | 0.00 | 15.00 | 25.00 | 10.00 | 0.00 | 6.50 | 0.00 |
| **330730** | Perfumery cosmetics or toilet preparations perfumed bath salts and other bath preparations | 5.00 | 5.35 | 15.00 | 25.00 | 20.00 | 0.00 | 6.50 | 0.00 |
| **340111** | Soap and organic surface-active products in the form of bars cakes moulded shapes and paper wadding felt and nonwovens impregnated coated or covered with soap or detergent for toilet use including medicated products | 5.00 | 0.00 | 20.00 | 25.00 | 20.00 | 0.00 | 6.50 | 0.00 |

| **Appendix. C: Results of the TINA simulation for USA** | | | | | | | | |
| --- | --- | --- | --- | --- | --- | --- | --- | --- |
| **HS 8-digit** | **Description** | **Bilateral Trade Before** | **Bilateral Trade After** | **Change** | **Trade Creation** | **Trade Diversion** | **Percentage Change** | **Effected Economies** |
| 330730 | Perfumery cosmetic or toilet preparations perfumed bath salts and other bath preparations | 545993 | 629876 | 83883 | 42499 | 41384 | 15.36 | 36 |

| **Appendix. D: Results of the TINA simulation for UAE** | | | | | | | | |
| --- | --- | --- | --- | --- | --- | --- | --- | --- |
| **HS 8-digit** | **Description** | **Bilateral Trade Before** | **Bilateral Trade After** | **Change** | **Trade Creation** | **Trade Diversion** | **Percentage Change** | **Effected Economies** |
| 330420 | Cosmetic and toilet preparations eye makeup | 606668 | 668876 | 62208 | 19113 | 43095 | 10.25 | 58 |
| 330499 | Cosmetic and toilet preparations nec in heading no 3304 for the care of the skin excluding medicaments including sunscreen or suntan preparations | 19415890 | 24915330 | 5499440 | 4138620 | 1360820 | 28.32 | 78 |
| 330590 | Hair preparations nec in heading no 3305 | 16953216 | 18736853 | 1783637 | 671944 | 1111693 | 10.52 | 54 |
| 330610 | Oral or dental hygiene preparations dentifrices | 13802583 | 14875174 | 1072591 | 162341 | 910250 | 7.77 | 29 |
| 330730 | Perfumery cosmetic or toilet preparations perfumed bath salts and other bath preparations | 171240 | 190953 | 19713 | 7599 | 12114 | 11.51 | 29 |
| 340111 | Soap and organic surfaceactive products in the form of bars cakes moulded shapes and paper wadding felt and nonwovens impregnated coated or covered with soap or detergent for toilet use including medicated products | 15918562 | 19353043 | 3434481 | 2463483 | 970998 | 21.58 | 58 |

| **Appendix. E: Results of the TINA simulation for Nepal** | | | | | | | | |
| --- | --- | --- | --- | --- | --- | --- | --- | --- |
| **HS 8-digit** | **Description** | **Bilateral Trade Before** | **Bilateral Trade After** | **Change** | **Trade Creation** | **Trade Diversion** | **Percentage Change** | **Effected Economies** |
| 300390 | Medicaments not containing antibiotics hormones alkaloids or their derivatives for therapeutic or prophylactic uses not packaged for retail sale | 24907596 | 27152492 | 2244896 | 2231160 | 13736 | 9.01 | 9 |
| 300490 | Medicaments consisting of mixed or unmixed products nec in heading no 3004 for therapeutic or prophylactic uses packaged for retail sale | 172074339 | 236050586 | 63976247 | 62418966 | 1557281 | 37.18 | 24 |
| 330420 | Cosmetic and toilet preparations eye makeup | 1003998 | 1595520 | 591522 | 487705 | 103817 | 58.92 | 23 |
| 330499 | Cosmetic and toilet preparations nec in heading no 3304 for the care of the skin excluding medicaments including sunscreen or suntan preparations | 25412212 | 39841465 | 14429253 | 62418966 | 1557281 | 56.78 | 24 |
| 330590 | Hair preparations nec in heading no 3305 | 13177669 | 31854207 | 18676538 | 18090366 | 586172 | 141.73 | 28 |
| 330610 | Oral or dental hygiene preparations dentifrices | 4914361 | 6902464 | 1988103 | 1950510 | 37593 | 40.45 | 14 |
| 330730 | Perfumery cosmetic or toilet preparations perfumed bath salts and other bath preparations | 39806 | 63596 | 23790 | 19371 | 4419 | 59.76 | 7 |
| 340111 | Soap and organic surfaceactive products in the form of bars cakes moulded shapes and paper wadding felt and nonwovens impregnated coated or covered with soap or detergent for toilet use including medicated products | 2472550 | 2564584 | 92034 | 5585 | 86449 | 3.72 | 23 |
|  |  |  |  |  |  |  |  |  |

| **Appendix. F: Results of the TINA simulation for Bangladesh** | | | | | | | | |
| --- | --- | --- | --- | --- | --- | --- | --- | --- |
| **HS 8-digit** | **Description** | **Bilateral Trade Before** | **Bilateral Trade After** | **Change** | **Trade Creation** | **Trade Diversion** | **Percentage Change** | **Effected Economies** |
| 300390 | Medicaments not containing antibiotics hormones alkaloids or their derivatives for therapeutic or prophylactic uses not packaged for retail sale | 5124174 | 6973725 | 1849551 | 1494220 | 355331 | 36.09 | 10 |
| 300490 | Medicaments consisting of mixed or unmixed products nec in heading no 3004 for therapeutic or prophylactic uses packaged for retail sale | 11096052 | 15740546 | 4644494 | 3777039 | 867455 | 41.86 | 23 |
| 330420 | Cosmetic and toilet preparations eye makeup | 166464 | 232090 | 65626 | 19838 | 45788 | 39.42 | 12 |
| 330499 | Cosmetic and toilet preparations nec in heading no 3304 for the care of the skin excluding medicaments including sunscreen or suntan preparations | 17630551 | 31713245 | 14082694 | 11341104 | 2741590 | 79.88 | 21 |
| 330590 | Hair preparations nec in heading no 3305 | 2299055 | 9040131 | 6741076 | 6330947 | 410129 | 293.21 | 14 |
| 330610 | Oral or dental hygiene preparations dentifrices | 3750277 | 4133096 | 382819 | 182803 | 200016 | 10.21 | 15 |
| 330730 | Perfumery cosmetic or toilet preparations perfumed bath salts and other bath preparations | 764368 | 912623 | 148255 | 135796 | 12459 | 19.4 | 11 |
| 340111 | Soap and organic surfaceactive products in the form of bars cakes moulded shapes and paper wadding felt and nonwovens impregnated coated or covered with soap or detergent for toilet use including medicated products | 1664724 | 2157869 | 493145 | 141356 | 351789 | 29.62 | 12 |

| **Appendix. G: Results of the TINA simulation for South Africa** | | | | | | | | |
| --- | --- | --- | --- | --- | --- | --- | --- | --- |
| **HS 8-digit** | **Description** | **Bilateral Trade Before** | **Bilateral Trade After** | **Change** | **Trade Creation** | **Trade Diversion** | **Percentage Change** | **Effected Economies** |
| 330420 | Cosmetic and toilet preparations eye makeup | 10779 | 14379 | 3600 | 907 | 2693 | 33.4 | 20 |
| 330499 | Cosmetic and toilet preparations nec in heading no 3304 for the care of the skin excluding medicaments including sunscreen or sun tan preparations | 1386284 | 1830605 | 444321 | 100335 | 343986 | 32.05 | 56 |
| 330590 | Hair preparations nec in heading no 3305 | 783259 | 1121930 | 338671 | 146629 | 192042 | 43.24 | 42 |
| 330610 | Oral or dental hygiene preparations dentifrices | 2539087 | 3490490 | 951403 | 619792 | 331611 | 37.47 | 21 |
| 330730 | Perfumery cosmetic or toilet preparations perfumed bath salts and other bath preparations | 6285 | 21927 | 15642 | 14073 | 1569 | 248.88 | 8 |
| 340111 | Soap and organic surfaceactive products in the form of bars cakes moulded shapes and paper wadding felt and nonwovens impregnated coated or covered with soap or detergent for toilet use including medicated products | 835033 | 1589733 | 754700 | 550594 | 204106 | 90.38 | 27 |

| **Appendix. H: Results of the TINA simulation for Russia** | | | | | | | | |
| --- | --- | --- | --- | --- | --- | --- | --- | --- |
| **HS 8-digit** | **Description** | **Bilateral Trade Before** | **Bilateral Trade After** | **Change** | **Trade Creation** | **Trade Diversion** | **Percentage Change** | **Effected Economies** |
| 300490 | Medicaments consisting of mixed or unmixed products nec in heading no 3004 for therapeutic or prophylactic uses packaged for retail sale | 361619799 | 594714537 | 233094738 | 214793101 | 18301637 | 64.46 | 36 |
| 330420 | Cosmetic and toilet preparations eye makeup | 61968 | 69211 | 7243 | 1574 | 5669 | 11.69 | 19 |
| 330499 | Cosmetic and toilet preparations nec in heading no 3304 for the care of the skin excluding medicaments including sunscreen or sun tan preparations | 1085302 | 1227411 | 142109 | 42915 | 99194 | 13.09 | 36 |
| 330590 | Hair preparations nec in heading no 3305 | 224832 | 300661 | 75829 | 55267 | 20562 | 33.73 | 30 |
| 330610 | Oral or dental hygiene preparations dentifrices | 3281332 | 3871284 | 589952 | 297907 | 292045 | 17.98 | 22 |
| 330730 | Perfumery cosmetic or toilet preparations perfumed bath salts and other bath preparations |  |  |  |  |  |  |  |
| 340111 | Soap and organic surfaceactive products in the form of bars cakes moulded shapes and paper wadding felt and nonwovens impregnated coated or covered with soap or detergent for toilet use including medicated products | 61939 | 70266 | 8327 | 3483 | 4844 | 13.44 | 17 |
